# Angiosarcoma of the pulmonary artery mimicking pulmonary thromboembolism

**DOI:** 10.4102/sajr.v25i1.2150

**Published:** 2021-08-10

**Authors:** Sitang Nirattisaikul, Arunee Dechaphunkul, Keerati Hongsakul

**Affiliations:** 1 Department of Radiology, Faculty of Medicine, Prince of Songkla University, Hatyai, Thailand; 2 Department of Internal Medicine, Faculty of Medicine, Prince of Songkla University, Hatyai, Thailand

**Keywords:** angiosarcoma, pulmonary thromboembolism, CT pulmonary angiography, pulmonary sarcoma, pulmonary artery

## Abstract

Primary pulmonary angiosarcomas (PPAs) are rare, and often, their diagnosis is delayed because of insidious clinical symptoms and imaging findings mimicking pulmonary thromboembolism (PE). A 33-year-old female patient presented with chest pain and progressive dyspnoea. Her initial diagnosis, based on clinical symptoms and CT pulmonary angiography (PA) findings, was PE. However, after treatment with anticoagulants, the patient failed to improve. A follow-up CTPA and further CT-guided biopsy results were compatible with angiosarcoma.

## Introduction

Primary pulmonary angiosarcoma (PPA) is a rare tumour with a poor prognosis. The rarity of this condition and the difficulty in distinguishing the clinical and imaging findings from pulmonary thromboembolism (PE) often result in a delayed diagnosis, usually with local invasion or metastases and therefore, a poor prognosis. At present, advanced imaging techniques such as computed tomography pulmonary angiography (CTPA) help improve the rate of detection and diagnostic accuracy of PPA. This report describes a case of PPA and reviews the literature on the imaging findings, using various modalities to distinguish between PPA and PE.

## Case

**FIGURE 1:**
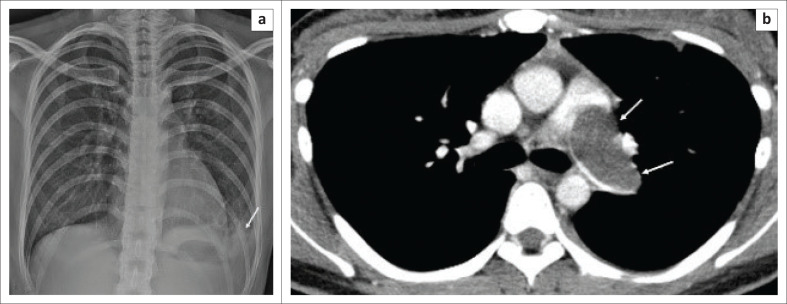
(a) Frontal chest radiograph demonstrates normal sized main, right and left pulmonary arteries with a small left basal pleural effusion (arrow). (b) Axial computed tomography pulmonary angiography demonstrated a filling defect extending from a dilated main pulmonary artery into the left pulmonary artery (arrows).

A 33-year-old female non-smoker presented with a 1-month history of cough, chest pain and progressive dyspnoea. She had no fever, haemoptysis or lower limb oedema. The frontal radiograph revealed normal sizes of the main, right and left pulmonary arteries with a minimal left pleural effusion (Figure 1a). Computed tomography pulmonary angiography was performed because of suspected PE, which demonstrated a poorly enhancing hypodense lesion involving a dilated left main pulmonary artery and pulmonary trunk and subpleural consolidation in the left upper lobe (Figure 1b).

**FIGURE 2:**
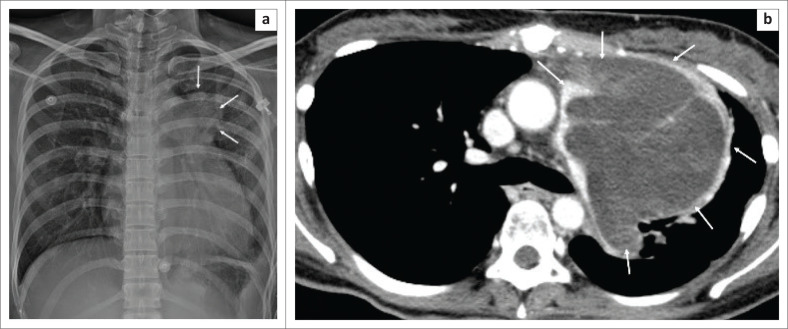
(a) Follow-up frontal chest radiograph revealed an enlarged main pulmonary artery and a left hilar mass (arrows). (b) Axial computed tomography pulmonary angiography demonstrated a large lobulated rim enhancing exophytic mediastinal mass extending from the dilated main pulmonary artery and the left pulmonary artery (arrows).

**FIGURE 3:**
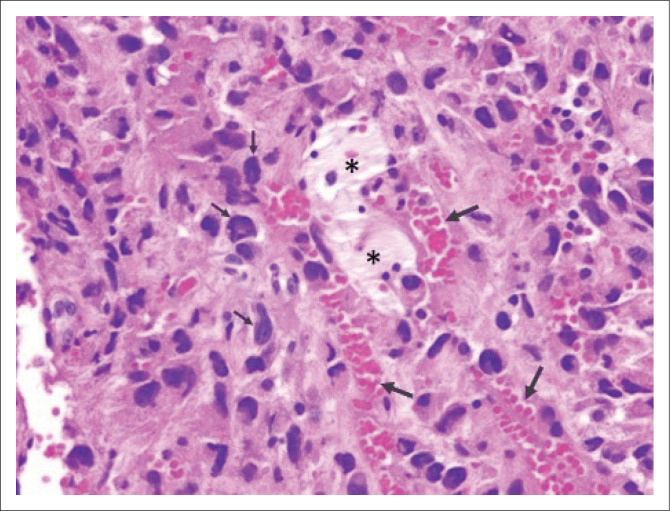
Microscopic examination demonstrated high-grade malignant cells (small arrows) with massive haemorrhage (large arrows) and necrosis (asterisks).

The patient was diagnosed with acute PE and pulmonary infarction in the left upper lobe. Although she was subsequently treated with anticoagulants, her clinical condition failed to improve. Follow-up frontal radiograph and CTPA was repeated three months after treatment. The frontal chest radiograph indicated new findings of an enlarged main pulmonary artery and a left hilar mass (Figure 2a). Computed tomography PA revealed progressive enlargement of the previous poorly enhancing lesion, now involving the right and left pulmonary arteries and main pulmonary trunk with extraluminal extension into the mediastinum (Figure 2b). Computed tomography-guided biopsy of the mediastinal lesion was performed. Histopathologic analysis of the specimen revealed high-grade malignancy with massive haemorrhage and necrosis, consistent with angiosarcoma (Figure 3). Unfortunately, the patient demised before completing treatment with chemoradiation therapy.

## Discussion

Primary sarcoma of the great vessels is a rare malignant tumour with less than 1% prevalence amongst all sarcomas, usually only documented in case reports and case series studies.^1,2^ It is more common in middle-aged patients with no sex predominance.^1^ Primary sarcoma of the pulmonary artery is classified by location into the intimal and mural types, with the intimal type more commonly encountered than the mural type. The most common locations of PPA are at the pulmonary artery trunk^3^ and proximal pulmonary artery. Unilateral involvement of the pulmonary vessels is frequently observed in PPA compared with embolic disease.^1,2^

Patients with PPA may present with clinical symptoms resembling those frequently seen in PE including dyspnoea, chest pain, cough and haemoptysis.^1^ Consequently, an initial diagnosis of PE is usually made, before lung cancer and even more rarely PPA. However, if clinical symptoms progress in the absence of predisposing factors for PE, PPA should be considered.

Imaging modalities used to diagnose PPA include CTPA, MRI, transthoracic echocardiography (TTE) and Positron emission tomography with 2-deoxy-2[fluorine-18]fluoro-D-glucose integrated with CT (^18^F-FDG PET/CT). However, definite diagnosis with imaging alone is difficult because of the imaging findings of PPA mimicking PE. Chest radiography may be normal or appear similar to pulmonary arterial hypertension or a hilar mass. Computed tomography pulmonary angiography is the initial modality of choice when PE is suspected. Both PPA and PE have similar cross-sectional imaging findings, usually a filling defect in the pulmonary artery. A retrospective review of the initial CTPA in this case revealed a poorly enhancing filling defect in the dilated left main pulmonary artery and pulmonary trunk, resulting in an initial misdiagnosis of PE. Imaging findings that may suggest the diagnosis of PPA over PE include a unilateral filling defect occupying the entire luminal diameter of the main or proximal pulmonary arteries, distension of the portion of the involved pulmonary artery, extraluminal extension of the tumour, heterogeneous enhancement and smooth vascular tapering without abrupt narrowing or cut-off as well as mosaic attenuation in the lung parenchyma and metastasis to the lung or distant organs.^1,2,4^ The attenuation of PPA on CTPA may be homogeneous, similar to bland thrombus in PE. Heterogeneous density could be related to areas of haemorrhage, necrosis or cyst formation within the tumour. Stable or progressive filling defects seen at follow-up CTPA after adequate anticoagulant therapy for PE may favour alternative diagnoses such as PPA.^2,5^ In the case presented, progressive enlargement of the previous filling defect was observed, with involvement of the dilated right and left pulmonary arteries and main pulmonary trunk and extraluminal extension into the mediastinum, all findings suggestive of pulmonary artery sarcoma. The diagnosis of PPA was confirmed at CT-guided biopsy and histopathological diagnosis of angiosarcoma.

Magnetic resonance imaging and ^18^F-FDG PET/CT are advanced imaging techniques used to diagnose PPA. MRI demonstrates high signal intensity on T2-weighted short-tau inversion recovery imaging (STIR) and is superior to CTPA in the evaluation of vascularisation seen as heterogeneous enhancement.^6^ ^18^F-FDG PET/CT helps distinguish between benign conditions and malignant tumours. In PPA, ^18^F-FDG PET/CT shows increased uptake because of hypermetabolic activity whilst negative uptake in PE.^2,7^ However, very few cases of ^18^F-FDG PET/CT for PPA have been performed thus far. At TTE, mass mobility, attachment to the pulmonary artery wall or pulmonary artery valve, absence of echolucent areas and bulging rather than linear morphology favour the diagnosis of PPA over PE.^6^

The primary management of PPA is surgical, and options include pulmonary endarterectomy, lobectomy and pneumonectomy and/or a combination of chemotherapy and radiation. However, the prognosis is poor with a median survival of 26 months after surgery.^8^

## Conclusion

This report describes a case of PPA where clinical and imaging findings overlapped with PE resulting in an initial misdiagnosis and therefore delayed treatment and poor prognosis. Although PPA is a rare disease, clinicians and radiologists should keep this differential diagnosis in mind when there is filling defect in an expanded pulmonary artery with chronic gradual progression of the disease, absence of risk factors for thromboembolism, lack of clinical improvement after adequate treatment with anticoagulants and lung metastases but no known primary or features of other primary malignancy. Computed tomography pulmonary angiography helps differentiate between PPA and PE, and pulmonary artery sarcoma should be considered when there is a heterogeneously enhancing lesion in a distended pulmonary artery with extraluminal extension. Moreover, the patient should return for an unenhanced scan to confirm enhancement if PPA is suspected.
